# Elimination of strength degrading effects caused by surface microdefect: A prevention achieved by silicon nanotexturing to avoid catastrophic brittle fracture

**DOI:** 10.1038/srep10869

**Published:** 2015-06-04

**Authors:** Kunal Kashyap, Amarendra Kumar, Chuan-Torng Huang, Yu-Yun Lin, Max T. Hou, J. Andrew Yeh

**Affiliations:** 1 Institute of Nanoengineering and Microsystems, National Tsing Hua University, No. 101, Section 2, Kuang-Fu Road, Hsinchu 30013, Taiwan; 2 Department of Civil Engineering, National Cheng Kung University, No.1, University Road, Tainan City 701, Taiwan; 3 Department of Mechanical Engineering, National United University, No.1, Lienda, Miaoli 36003, Taiwan; 4 Department of Power Mechanical Engineering, National Tsing Hua University, No. 101, Section 2, Kuang-Fu Road, Hsinchu 30013, Taiwan; 5 Instrument Technology Research Center, National Applied Research Laboratories, 20, R&D Road VI, Hsinchu Science Park, Hsinchu 30076, Taiwan

## Abstract

The unavoidable occurrence of microdefects in silicon wafers increase the probability of catastrophic fracture of silicon-based devices, thus highlighting the need for a strengthening mechanism to minimize fractures resulting from defects. In this study, a novel mechanism for manufacturing silicon wafers was engineered based on nanoscale reinforcement through surface nanotexturing. Because of nanotexturing, different defect depths synthetically emulated as V-notches, demonstrated a bending strength enhancement by factors of 2.5, 3.2, and 6 for 2-, 7-, and 14-μm-deep V-notches, respectively. A very large increase in the number of fragments observed during silicon fracturing was also indicative of the strengthening effect. Nanotextures surrounding the V-notch reduced the stress concentration factor at the notch tip and saturated as the nanotexture depth approached 1.5 times the V-notch depth. The stress reduction at the V-notch tip measured by micro-Raman spectroscopy revealed that nanotextures reduced the effective depth of the defect. Therefore, the nanotextured samples were able to sustain a larger fracture force. The enhancement in Weibull modulus, along with an increase in bending strength in the nanotextured samples compared to polished single-crystal silicon samples, demonstrated the reliability of the strengthening method. These results suggest that this method may be suitable for industrial implementation.

Defects inevitably exist in all materials regardless of how they are prepared. These defects significantly degrade material strength. From a practical standpoint, achieving the theoretical limit of material strength due to pre-existing, micro-sized manufacturing defects that cause ductile or brittle fracture under applied load is impossible^1^,^2^,^3^,^4^. Over the past few decades, researchers have explicitly addressed the causes and remedies of fracture for various material types, either brittle or ductile, to achieve the closest possible theoretical limit of material strength. However, these efforts have been limited because defect-free manufacturing processes are not available. Compared to ductile materials, brittle materials are more susceptible to sudden fracture because of stress concentration and crack propagation arising from defects^5^,^6^,^7^.

Silicon, which is extensively used in very large scale integrated (VLSI) circuits, nano/microelectromechanical systems (N/MEMS), and photovoltaic products, is quite brittle and suffers from catastrophic fracture due to concentrated stress at microdefects^8^,^9^,^10^ on its surface. These microdefects are generated because of external loading^8^, thermal processes^10^,^11^ or film growth^12^,^13^. Silicon wafer manufacturing includes several processing steps that are extensively used in industry, each of which multiplies the probability of surface defect formation. These steps include wafer handling^8^, back-side grinding^14^, chemical mechanical polishing (CMP)^15^, and dicing^16^. Each of these steps generates microdefects, such as scratches, dimples, mounds, and hazes. These undesirable features degrade wafer strength by localizing stress concentrations associated with crack initiation, which is responsible for wafer fracture.

Fulfilling the demands for silicon wafers to have an increased diameter and reduced thickness requires precise control of defects. This requirement has led numerous researchers to investigate various methodologies, such as crack deflection through implanting external elements into the bulk^17^ or reducing defect size^15^, for strengthening silicon wafers. Dopant diffusion by oxygen^18^, nitrogen^19^,^20^,^21^, and germanium^22^ has been shown to generate a greater pinning effect on dislocations, which delocalizes the stress concentration at defects and obstructs crack propagation. In a doped material, some of the original Si–Si bonds are replaced with higher-energy bonds that eventually strengthen the material^23^. However, the doping concentration changes the resistivity of the materials and influences the bulk properties of the silicon, which may be detrimental. Methods such as CMP^24^ and annealing the silicon wafer in oxygen^25^ reduce defect size while retaining bulk properties. Unavoidable large particles in the incoming slurry and agglomerated slurry on the polishing pad are the most likely causes of micro-scratches generated during the CMP process. This process can be controlled to an extent through process optimization, but optimization cannot effectively eliminate the presence of microdefects^26^.

Recently, nanoscale reinforcements for material strengthening have been developed for ceramics and glass. These methods have demonstrated a pronounced strengthening capability. Nanocoatings form brittle matrices and fibres to deflect the crack propagation pathways of ceramic materials^27^,^28^,^29^. Nanoparticles in the coating material on a glass tend to migrate into surface defects and reduce the stress concentration, thus improving the glass’ mechanical properties^30^,^31^. Most nanoscale reinforcement techniques fail to retain the bulk properties of the material. Approaches for improving the mechanical properties of silicon materials have not been sufficiently researched from the perspective of nanoscale reinforcement, and considerable material loss due to fracture still occurs in semiconductor processing. In our previous study, we demonstrated that nanotexturing on the surface of single-crystal silicon (sc-silicon) substrates could substantially enhance the strength of the material by a factor of six while maintaining its bulk properties^32^. The results of this previous study led us to conduct an in-depth macroscopic and microscopic exploration to elucidate the mechanism involved in the nanotexturing process.

## Results

**Figure 1:**
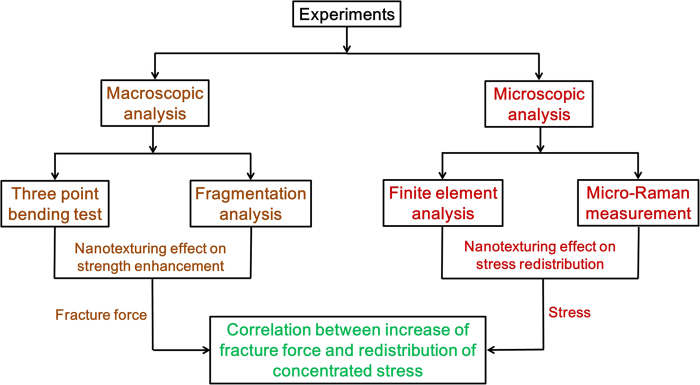
Illustration of the systematic investigation performed to elucidate the mechanism involved in strengthening silicon through nanotexturing.

Defects are randomly distributed on the surface of silicon, and predetermining which particular defect will result in fracture is difficult because of extremely fast crack initiation and propagation. In the present study, we evaluated the strengthening induced by surface nanotexturing through the introduction of a dominant V-notch defect in sc-silicon samples. The V-notch emulated a synthetic defect that functions as a single-stress concentrator that dominates all other defects and serves as a crack initiation point^13^,^33^,^34^,^35^,^36^. Fig. 1 illustrates the systematic investigation we performed to elucidate the mechanism through which nanotexturing strengthens silicon. The macroscopic-level investigation, in which we applied force to observe the bending strength and fragmentation behaviour in relation to V-notch depth, validated the strengthening capability of silicon nanotexturing. The macroscopic-level findings prompted us to extend the study into the microscopic level to investigate stress distribution through numerical simulation. We employed the finite element method and performed experimental measurements using micro-Raman spectroscopy. The information on fracture force was obtained from macroscopic analysis and subsequently coupled with the stress information obtained from microscopic analysis to further evaluate the underlying mechanism. We also demonstrated a rollable silicon wafer, achieved by applying this new strengthening technology, that could be useful for generating flexible and bendable electronics^37^,^38^,^39^ in the future.

### Sample fabrication and description

**Figure 2:**
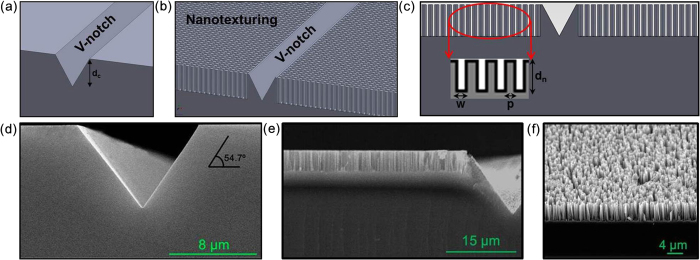
Structural description of samples. Schematic of **(a)** V-notch, **(b)** and **(c)** nanotextured V-notch. **(d)** and **(e)** SEM images of a non-textured V-notch and nanotextured V-notch sc-silicon sample, respectively. **(f)** SEM image of surface nanotexturing.

Two types of samples, including non-textured and nanotextured V-notch samples as shown in Figs. 2(a–c), were prepared to study the interaction between nanotextures and defects. The V-notch shown in Fig. 2(d) was fabricated through anisotropic tetramethylammonium hydroxide (TMAH) etching. As shown in Figs. 2(e,f), wet-chemically etched silicon nanotextures with a pitch (p) and a width (w) of 100 ± 10 nm were fabricated over the entire surface surrounding the V-notch using electroless metal deposition with Ag^+^ ions in an etchant composed of 4.6 M hydrofluoric acid (HF) and 0.02 M silver nitrate (AgNO_3_). The precipitated Ag dendrites formed as etching byproducts were removed with an aqueous solution of 70% concentrated nitric acid (HNO_3_)^40^,^41^. The morphology of the silicon nanotextures was controlled by fabricating a smooth nanotexture bottom, which may influence the strength and fracture of the samples (see Fig. S1 in supplementary material).

### Macroscopic analysis for strength enhancement

**Figure 3:**
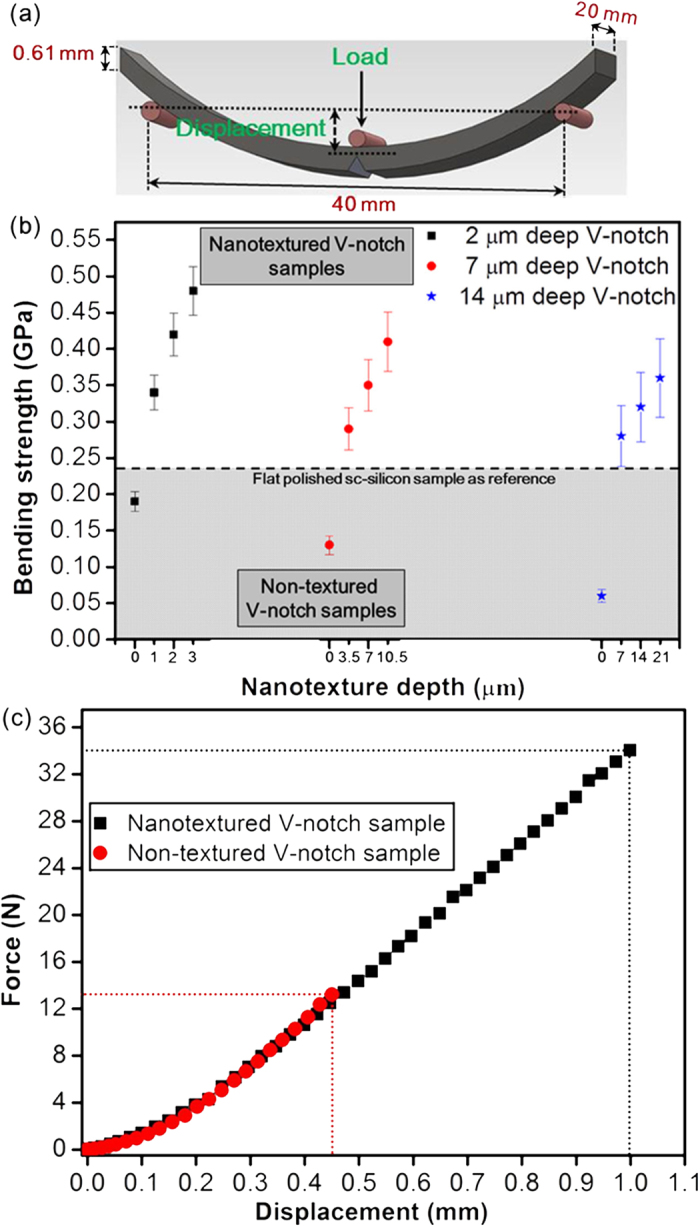
Macroscopic analysis of bending-strength measurements. **(a)** Sample description and schematic of the 3PB test. **(b)** The shaded region shows the bending strength of non-textured V-notch samples, and the white region shows the bending strength of nanotextured V-notch samples. The bending strength was enhanced from 0.19 GPa to 0.48 GPa (by approximately 2.5-fold) for a 2-μm-deep V-notch, from 0.13 GPa to 0.41 GPa (by approximately 3.2-fold) for a 7-μm-deep V-notch, and from 0.06 GPa to 0.36 GPa (by approximately 6-fold) for a 14-μm-deep V-notch at nanotexturing depths 1.5 times as deep as the V-notch depth. (**c**) Load displacement curves for non-textured and nanotextured (at a depth of 3.5 μm) 7-μm-deep V-notch samples.

Macroscopic measurements, including bending-strength analysis, were performed to evaluate the effects of increasing nanotexture depth (d_n_) on a given notch depth (d_c_). The samples were 60 mm in length, 20 mm in width, and 0.61 mm in thickness, satisfying the ASTM E 855-90 standard^42^ for the three-point bending (3PB) test (Fig. 3 (a)). The test was performed at a loading rate of 30 mm/min. The bending strength σ_*br*_ was determined by equation (1)^42^:
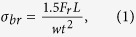
where *F*_*r*_ is the load at rupture and *L*, *w*, and *t* are span length, width, and thickness of the sample, respectively. A bending strength of 0.24 GPa for flat, polished sc-silicon samples was used as a reference and is shown by the black dotted line in Fig. 3(b). The microdefect depth of the silicon wafer was generally within the range of 1–8 μm^43^,^44^,^45^,^46^. Three different controllable depths of synthetic defects were added to the midpoint of the samples. The V-notch depths of 2, 7, and 14 μm were designed to validate the possibility of nanotexture strengthening for the entire range of microdefect depths generated during the wafer-processing steps. Although the depths of microcracks on the surface of polished sc-silicon samples is on the order of several microns, different samples may contain microcracks of different depths at various locations. The V-notch represents a major crack of controllable depth at the midpoint, and this defect supersedes all other defects. The stress concentration increased as the defect depth increased^47^, resulting in degradation of bending strengths to 0.19 GPa, 0.13 GPa, and 0.06 GPa for the 2-, 7-, and 14-μm-deep V-notch samples, respectively. The depth of V-notch was increased to 7 μm and 14 μm for ensuring the dominance of V-notch on other pre-existing defects. Samples with different nanotexture depths for each V-notch depth were fabricated to evaluate the effect of nanotexturing depth on strengthening. The bending strength of nanotextured V-notch samples was enhanced from 0.19 GPa to 0.48 GPa (2.5-fold) for 2-μm-deep V-notches, from 0.13 GPa to 0.41 GPa (3.2-fold) for 7-μm-deep V-notches, and from 0.06 GPa to 0.36 GPa (6-fold) for 14-μm-deep V-notches (Fig. 3(b)). For all V-notch depths, the bending strength increased with increasing nanotexture depth. The results indicate that nanotexturing ceases crack initiation from the notch tip and allows the silicon to sustain larger forces before fracture. The bending strength enhanced by nanotexturing of the V-notched samples was also insensitive to loading rate under quasistatic conditions (see supplementary Fig. S2).

The force displacement curves from the 3PB tests for the non-textured and nanotextured V-notch samples are shown in Fig. 3(c). All samples demonstrated very similar responses, which included nonlinear behaviour near the starting point, a linear region, and sudden fracture at the end. Insufficient friction between the fixture support and the sample resulted in the nonlinear behaviour due to slipping from the initial load. The stiffness (i.e., the slope of the force-displacement curves), given as 39.5 and 39 kN/m, was translated into Young’s modulus values of 169.8 GPa and 167.3 GPa for non-textured and nanotextured V-notch samples, respectively (see the Methods section for additional details). These values are similar to the reported value of 169 ± 7 GPa^48^. The small changes in the Young’s modulus for nanotextured samples also signifies that the bulk properties of the silicon were preserved.

**Figure 4:**
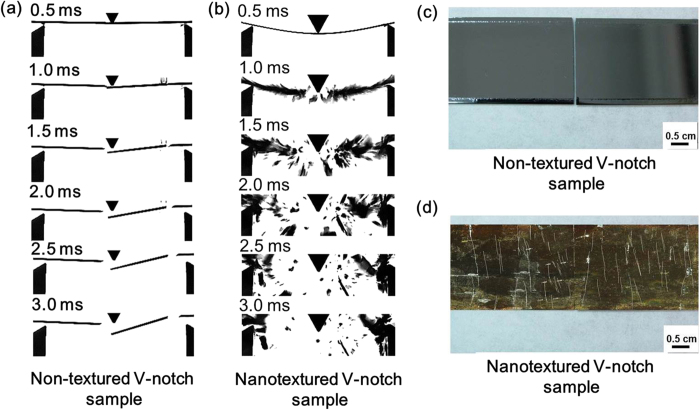
Comparison of fragmentation during fracture for a 7-μm-deep non-textured V-notch sample and a nanotextured (approximately 10.5 μm deep) 7-μm-deep V-notch sample. **(a)** and **(c)** A non-textured V-notch sample composed of two fragments just after fracture. **(b)** and **(d)** A nanotextured V-notch sample composed of multiple fragments immediately after fracture. The nanotextured sample in **(b)** sustained a larger force and deflection prior to fracture compared to the non-textured sample in **(a)**, demonstrating the improved strength and flexibility of the nanotextured silicon substrate.

The dynamic response of the fracture in the 3PB test was also observed using a high-speed camera. Fragmentation analysis is another direct macroscopic method for examining the strengthening mechanism. For any type of brittle material, a greater number of fragments produced during fracture will result in a greater sample strength due to high absorption of strain energy^49^. The non-textured V-notch samples broke neatly into two main pieces under an applied load, as shown in Figs. 4(a,c), because the V-notch suppressed the effects of all other pre-existing defects and functioned as a dominant crack initiation point. Fracture of the V-notch samples into only two fragments illustrates a reduction in material strength due to the V-notch. The nanotextured V-notch samples broke into several fragments during the fracture process, as shown in Figs. 4(b,d), which indicates the strength of the samples had been enhanced. The fragmentation of flat polished sc-silicon samples during fracture was also compared to that of the nanotextured V-notch samples (see Fig. S3 in supplementary material). The flat polished sc-silicon samples broke into a larger number of fragments compared to the non-textured V-notch samples and into a smaller number of fragments compared to the nanotextured V-notch samples. This result suggests that the strength of the samples was degraded by the V-notch and that the nanotextured samples were stronger compared to the non-textured samples or the flat polished sc-silicon samples.

Macroscopic observations confirmed that tuning the depth of nanotexturing for a defect size can effectively increase the strength of a material above the non-defective baseline strength. The overall phenomenon indicates the existence of some mechanism connecting the nanotexture to defects. The fragmentation results indicated that the V-notch induced a large concentration of stress at the notch tip compared to other regions and functioned as the exclusive crack initiator. The multiple fragments after fracture from the nanotextured samples might have been generated by the elimination of concentrated stress at the notch tip, which would likely have reduced the effective depth of the V-notch. To validate this hypothesis and to elucidate the mechanism, we conducted an in-depth microscopic-level investigation to analyse the stress behaviour.

### Microscopic analysis of stress distributio**n**

**Figure 5:**
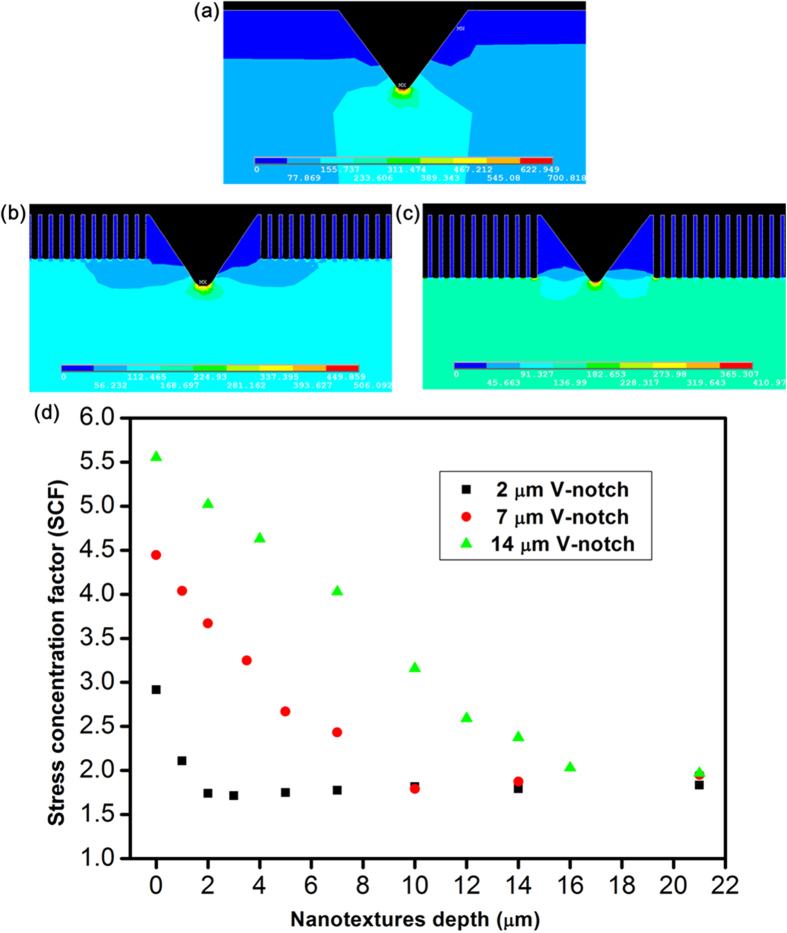
FEA simulation results of the 3PB test model for a sample 60 mm in length, 20 mm in width, and 0.61 mm in thickness under an applied force of 31 N. **(a)**–**(c)** Stress distribution of a 2-μm-deep V-notch with nanotexture depths of 0, 1, and 2 μm, respectively. **(a)** V-notch sample experiencing a maximum stress of 0.7 GPa concentrated at the notch tip. **(b)** Nanotextures of half the depth of the V-notch (d_n_ = 0.5d_c_) experiencing a reduced maximum stress of 0.5 GPa as a result of distributing stress throughout the nanotextured region. **(c)** Nanotextures of the same depth as the V-notch (d_n_ = d_c_) experiencing a reduced maximum stress of 0.4 GPa because of increased stress distribution in the nanotextured region. **(d)** A graph summarizing the reduction of the stress concentration factor depending on the depth of nanotexturing for 2-, 7-, and 14-μm-deep V-notch samples.

The stress behaviour was studied by simulating non-textured and nanotextured V-notch samples in a 3PB model using the ANSYS 12.1 (finite element analysis) software. Structural discontinuities, such as defects, are considered to induce localized stress, resulting in a large increase in the stress above a nominal value derived from completely defect-free samples (which are practically unachievable) under an applied force. The effects of a stress inducer can be determined in terms of a stress concentration factor (SCF) by which the stress of the region local to the discontinuity is increased over the nominal stress. The V-notch on the surface concentrated the stress at the notch tip (Fig. 5(a)), representing a condition suitable for evaluating the SCF. The factor SCF was defined as the ratio of the maximum stress (*σ*_*max*_) to the nominal stress (*σ*_*nom*_), as expressed in equation (2)^47^:
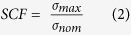


The nominal stress (*σ*_*nom*_) refers to the maximum stress applied to a flat polished sc-silicon substrate. An average fracture force of 31 N was experimentally evaluated by the 3PB test for flat polished sc-silicon samples, and this value for *σ*_*nom*_ was applied to all the simulation scenarios. The nominal stress was uniformly distributed over the flat silicon sample (see Fig. S4 in supplementary material); however, the presence of the V-notch concentrated and increased the stress in a small region near the notch tip to a level above the nominal value of stress. Consequently, no stress appeared at the surface near the V-notch, as shown in Fig. 5(a). For a nanotexture depth of zero, simulated samples with V-notch depths of 2, 7, and 14 μm exhibited SCF values of 2.9, 4.4, and 5.6, respectively, as shown in Fig. 5(d). These findings agree with the observation that SCF increases along with increasing V-notch depth^47^ and is responsible for the observed reduction in bending strength. As shown in Fig. 5(b), nanotexturing at half the depth of the V-notch initiated a reduction in stress at the notch tip and enhanced stress distribution in the nanotexture region. The extent of stress reduction and distribution increased when the nanotexture depth approached the depth of the V-notch, as shown in Fig. 5(c). This result implies that the effective depth of the V-notch was reduced until the effect of the V-notch was completely eliminated. Figure 5(d) shows that the SCF decreased to approximately 1.8 and saturated as the nanotexture depth approached 1.5 times the V-notch depth. The stress decreased at the notch tip and simultaneously increased in the nanotextured region near the V-notch as the depth of nanotexturing increased, which implies a redistribution of stress at the notch tip. Our finite element analysis results revealed two significant regions—the notch tip and the nanotextured region in the vicinity of the V-notch—worthy of further exploration through real-time stress measurements using micro-Raman spectroscopy.

**Figure 6:**
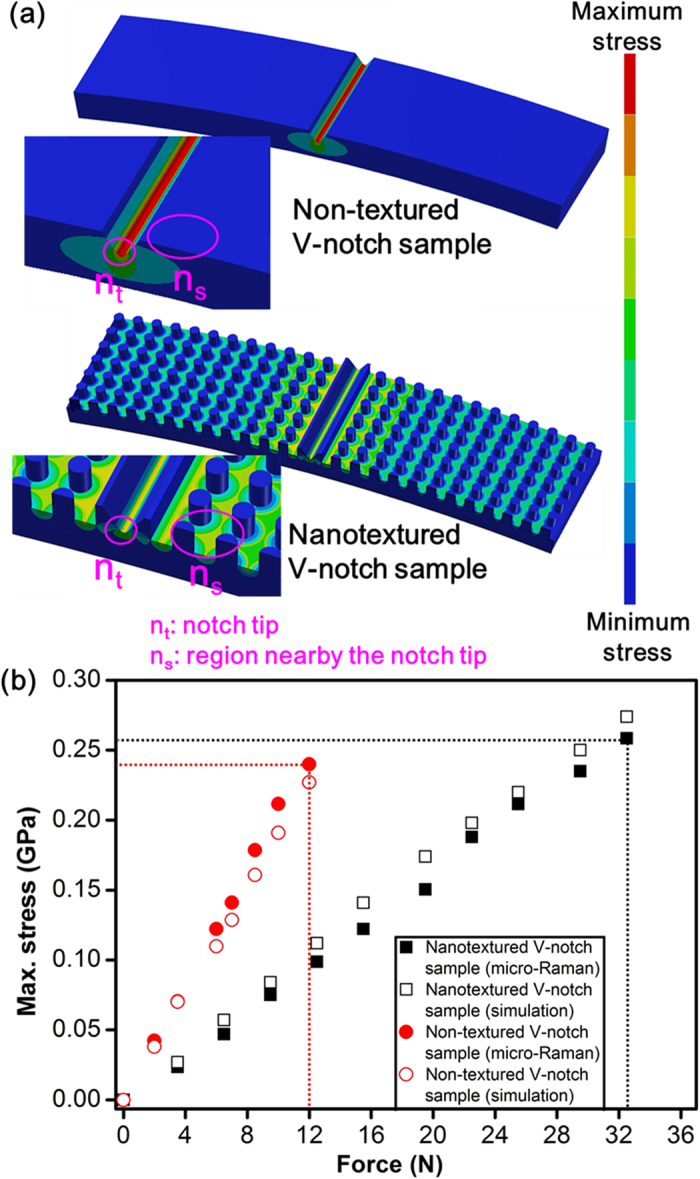
Micro-Raman analysis of a 7-μm-deep V-notch sample using Lorentzian curve fitting to elucidate the mechanism of stress redistribution. **(a)** Schematics of stress distributions representing the notch tip (n_t_) and the region near the V-notch (n_s_) for stressed and non-stressed silicon. **(b)** The variation in maximum stress generated at the notch tip with applied force explains the mechanism behind the nanotexture strengthening of V-notch samples. Non-textured V-notch samples endured fracture at a force of 12 N, whereas nanotextured V-notch samples sustained a larger force of 32.4 N while generating nearly the same amount of stress (approximately 0.26 GPa) at the notch tip.

The stress distribution is schematically shown in Fig. 6(a). Major stress appeared at the notch tip (n_t_) and at the region nearby the V-notch (n_s_). The stress was measured by micro-Raman spectroscopy under the 3PB condition to evaluate changes in stress distribution due to nanotexturing (refer to the supplementary material for the experimental setup, Fig. S5). Curve-fitting approximations using a Lorentzian function^50^ (refer to the methods section and supplementary Fig. S6) were generated to obtain the wavenumber shift (*Δω*), which can be converted into tensile stress (*σ*) for sc-silicon using equation (3)^50^:
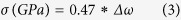
With no load applied, both non-textured and nanotextured V-notch samples measured at the notch tip retained the Raman peak at approximately 520.6 cm^−1^ (Figs. S6(a,c)), which is similar to the reported value for a non-stressed silicon peak^51^. The fracture force measured for a 7-μm-deep V-notch with and without nanotexturing was 34 N and 13.2 N, respectively. The Raman spectra were obtained just prior to fracture at 32.4 N and 12 N of force for the non-textured and nanotextured samples, respectively. These values were close to the fracture force. Under the 3PB condition, the peak shifted by 0.53 cm^−1^ to lower wavenumbers under the applied load of 12 N for the non-textured V-notch sample and by 0.55 cm^−1^ under 32.4 N for the nanotextured V-notch sample (Figs. S6(b,d)). The Raman spectrum was also captured for the nanotextured region near the V-notch (n_s_), and the wavenumber shifted by 0.11 cm^−1^ under an applied load of 12 N for the non-textured V-notch sample and by 0.32 cm^−1^ under 32.4 N for the nanotextured V-notch sample. The generated stress corresponding to wavenumber shift was determined by equation (3). Fig. 6(b) demonstrates that a larger fracture force was endured by the nanotextured V-notch samples. The mechanism can be explained by observing the behaviour of generated stress with increments in the force for both non-textured and nanotextured V-notch samples. The non-textured V-notch sample generated a maximum sustainable stress of 0.24 GPa (at notch tip) after a force of 12 N was applied; however, the nanotextured V-notch sample generated 0.1 GPa (at notch tip) after the same 12 N of force was applied. These results indicate that the larger fracture force of 34 N was accommodated while generating nearly the same maximum sustainable stress of 0.26 GPa. Simultaneously, the nanotextured region began distributing stress from the notch tip as soon as the force was applied, which was evident from the observed increase in stress from 0.05 GPa (for non-textured V-notch samples) to 0.15 GPa (for nanotextured V-notch samples) in the region near the V-notch (n_s_) just prior to fracture (Fig. S6(e)). This result implies that nanotexturing the V-notch samples reduced the effective defect depth, which enabled the samples to accommodate a larger force and led to the same amount of stress at the notch tip. These results agree with the fact that the mechanical strength of brittle materials can be improved by reducing defect depth through reduction in stress generated by an applied force^47^.

## Discussion

**Figure 7:**
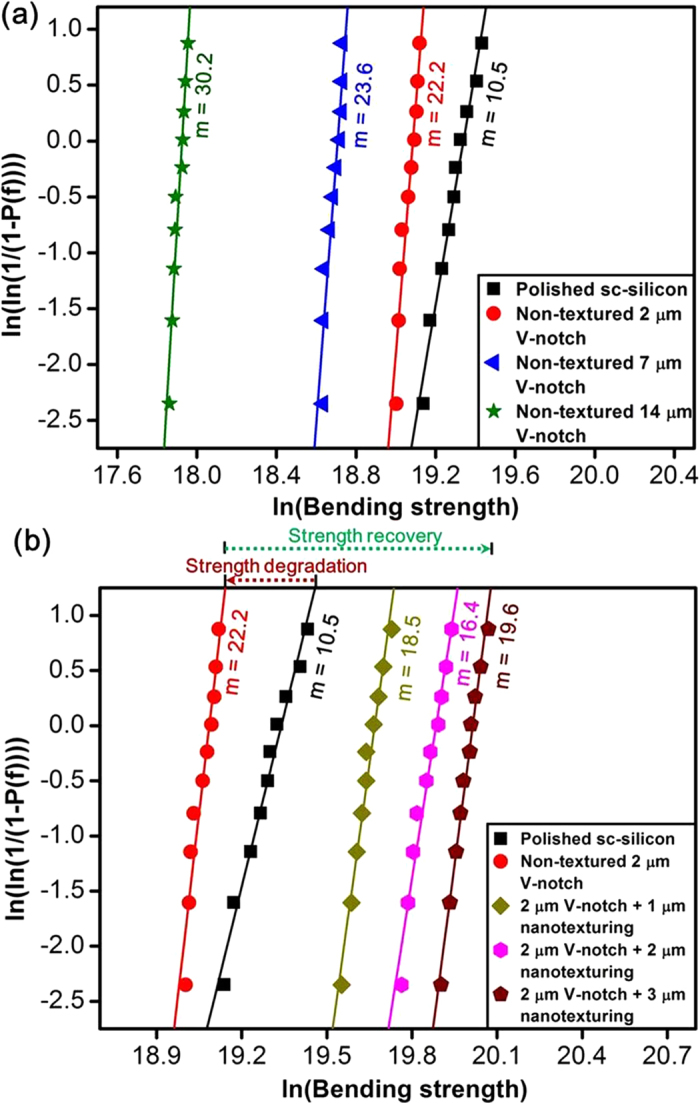
Weibull analysis to explain the strengthening effect and the reliability of nanotexturing methodology. **(a)** Comparison of the Weibull modulus (*m*) for the non-textured V-notch samples with different depths and polished sc-silicon samples; this comparison shows the predominance of the V-notch over other pre-existing defects. **(b)** Enhancement of the strength and reliability resulting from nanotexturing in comparison to the strength and reliability of the polished sc-silicon samples.

The modest enhancement in strength achieved by nanoscale reinforcement is now possible for silicon. The macroscopic 3PB test was coupled with micro-Raman stress measurements to establish a correlation between macro and micro measurements for investigating the strengthening mechanism. The enhanced strength along variations in a series of measured bending strengths under identical testing conditions was also essential and was analysed through Weibull estimation. The presence of unpredictable, randomly distributed micro-defects in polished sc-silicon samples cause large variations in bending strength, which is undesirable. The Weibull modulus (*m*) is a parameter that defines statistical variations in measured bending strength of nominally identical brittle-material samples under the same testing conditions (larger variations result in a lower value of *m*) based on the probability of failure (P_f_)^52^,^53^ (see the Methods section for more details of Weibull estimation). A statistical analysis of bending strength measured by a 3PB test was performed. The Weibull modulus (*m*) estimated for polished sc-silicon samples was 10.5, which was comparatively lower than the non-textured V-notch samples measured as 22.2, 23.6, and 30.2 for 2-, 7-, and 14-μm depths of the V-notch, respectively (Fig. 7(a)). Predicting the dominant defect (responsible for fracture) among pre-existing defects is difficult and varies from sample to sample for polished sc-silicon, leading to a relatively low value of m. The large value of m for non-textured V-notch samples ensured that the intentionally created V-notch defect was predominant over other pre-existing defects. The value of m increased, but the bending strength decreased as the V-notch depth increased (Fig. 7(a)). This result demonstrated that the inevitability of the crack initiation from the notch tip increases with V-notch depth and further justifies that the non-textured V-notch samples fractured into two fragments because of the presence of large stress concentration under an applied load at the notch tip.

The bending strength of 2 μm for non-textured V-notch samples was similar to that of the polished sc-silicon samples and was further analysed by Weibull estimation (refer to the supplementary material for the Weibull estimation of the 7- and 14-μm-deep V-notch samples, Fig. S7). Nanotexturing not only recovered and enhanced the bending strength but also increased the Weibull modulus (m) compared to that of the polished sc-silicon samples (Fig. 7(b)). The Weibull modulus (m) measured for 1 μm, 2 μm, and 3 μm of nanotextures of the 2 μm V-notch samples was 18.5, 16.3, and 19.6, respectively. Nanotexturing reduced the large scattering in a series of measured bending strengths from identical samples, which enhanced both the ‘m’ and strength compared to those of polished sc-silicon samples. The Weibull modulus was improved because of nearly uniform nanotexturing over the surface, which prevented any non-predictable pre-existing defects from becoming dominant (also demonstrated by the occurrence of multiple fragments during fracture) and demonstrated the reliability of the methodology.

**Figure 8:**
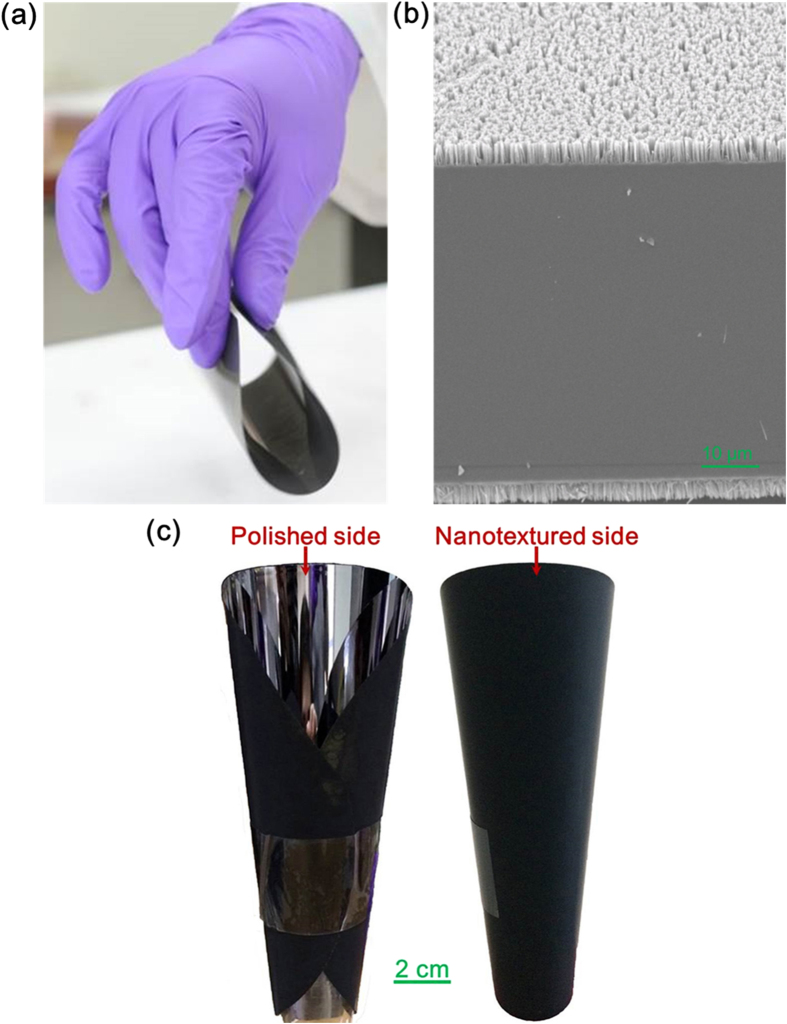
Visualization of a 6-inch-diameter, 70 ± 5-μm thick, high-strength rollable/foldable silicon wafer (see supplementary video). **(a)** Rollable silicon wafer with nanotexturing on both surfaces. **(b)** SEM image of 5-μm-deep nanotextures on both sides of the wafer. **(c)** Rollable silicon wafer with nanotexturing on one surface, whereas the other surface remains polished.

We conclude that silicon nanotextures with a pitch and a width of 100–150 nm and a depth greater than the pre-existing manufacturing defect depth can effectively enhance the strength of silicon wafers. A nanotexturing depth of 5–10 μm, depending on the wafer type, could eliminate the strength-degrading effect of defects. The 3PB tests revealed enhanced material strength, resulting in a larger displacement before fracture for nanotextured V-notch samples compared to non-textured samples (Fig. 3(c)). The results suggest that nanotexturing can enhance the strength of silicon materials such that less fragile silicon substrates with thicknesses below 100 μm are now possible. Silicon wafers with a 70 ± 5 μm thickness incorporating nanotextures at a 5 μm depth are feasible for mass production of rollable wafers, shown in Figs. 8(a,b). Generally, the backsides of silicon substrates used in semiconductor manufacturing industries do not contain electronic circuitry. Here, we also present a rollable silicon wafer in Fig. 8(c) with nanotexturing on the backside and a polished surface on the front side for semiconductor processing (see the supplementary video). These wafers would allow for manufacturing of flexible silicon solar cells with thicknesses less than 100 μm and of RF devices with thicknesses less than 50 μm. This technology need not be limited solely to flexible and bendable electronics. In the future, we believe that the backside of silicon substrates, which is generally not used for any purpose, could be nanotextured to enhance strength and control for material loss due to fracture, thereby reducing manufacturing costs. The semiconductor, photovoltaic, and MEMS industries suffer from yield losses due to mechanical failure, and these industries could all benefit from this technology.

## Methods

### Specimen preparation

A dry silicon oxide 250-nm-thick (hard mask for TMAH etching) was formed on n-(100) sc-silicon wafers (1–10 Ω·cm resistivity). The wafers were diced along the Si (110) direction with a sawing machine (Disco DAD 2 H/6 T) at a dicing rate of 5 mm/s, producing samples of 60 mm (length) × 20 mm (width) × 0.6 mm (thickness). The slow dicing rate was maintained to reduce the damage layer produced at the edges and sidewalls of the diced samples. The produced damage layers were etched by HNA composed of HF (49%), HNO_3_ (70%), and CH_3_COOH (99%) in a volume ratio of 2:7:1^54^. The top and bottom surfaces of the samples were protected by anti-acid tape to avoid undesirable acidic and alkali attack on the surfaces. The sidewall and edges (<1 mm) were exposed to the HNA etching to remove the damage layer of 50-80 μm produced by dicing. The samples with protective tape were submerged in acetone solution and isopropyl alcohol to remove the protective tape and subsequently cleaned with DI water. The samples were photolithographically patterned and then chemically etched with a 25% concentrated TMAH and IPA (isopropyl alcohol) solution at 85 °C to fabricate the V-notch. The IPA was mixed with TMAH to reduce the surface roughness during the anisotropic etching of the V-notch. The TMAH etching time depended upon the photolithographically obtained pattern width of 3, 10, and 20 μm specifically designed for 2-, 7-, and 14-μm-deep V-notches, respectively. The hard mask of SiO_2_ was removed by buffer oxide etching (BOE), which also removed residues from the anti-acid protection tape, to obtain the most immaculate samples possible for the nanotexturing process. Electroless metal deposition etching was performed using 4.6 M HF and 0.02 M AgNO_3_ that produced silicon nanotextures^26^ on the samples, except for the V-notch, which was covered with polymer. The residual pattern formed as Ag dendrites were removed with an aqueous solution of 70% concentrated nitric acid (HNO_3_) (refer to the supplementary material for the complete process, Fig. S8).

### Macroscopic analysis using the 3PB test

The sample was placed under the material testing machine (Hung Ta 8336) at room temperature using a load cell that yields to rupture at a displacement rate of 30 mm/min by the load applicator. The support span for testing was 40 mm × 20 mm × 0.6 mm (Fig. 3(a)) to minimize the formation of a sliding boundary and nonlinear stiffness under a smaller load. According to the linear elastic mechanism, the bending strength and Young’s modulus can be determined from the 3PB test^25^. The stiffness measured from the slope of the force-displacement curves (Fig. 3(c)) was translated into the Young’s modulus (*E*) by equation (4)^42^:
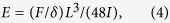
where *F* is the applied force, *δ* is the displacement, *I* is the moment of inertia for the sample (3.1 × 10^−13^ m^4^), *L* is the span length, and (*F/δ*) is the stiffness.

### Dynamic fracture behaviour observed by high-speed camera

The rupture photographs of non-textured and nanotextured V-notch samples were captured using a high-speed camera (IDT Y-4) recording at a frame rate of 2,000 frames/s under the illumination of a 500-W halogen lamp with a 950-μs exposure time and 1,280 × 1,024 pixel resolution. The camera was equipped with a lens (TAMRON A09N) with a focal length of 30 cm. To better illustrate the detailed fracture phenomena, the fracture of the polished sc-silicon sample was also compared to that of the nanotextured sample (see supplementary Fig. S3).

### Microscopic characterization by micro-Raman spectroscopy for stress analysis

A micro-Raman spectroscopy system (Jobin-Yvon T64000) was integrated with the 3PB testing machine and a thermoelectrically cooled charge-coupled device, as shown in the supplementary material (Fig. S5). Line scanning was conducted at each applied force by moving the sample stage for focusing the laser spot within the V-notch and in the nearby nanotextured region. The measurement technique employed a linearly polarized neodymium-doped yttrium aluminium garnet (Nd:YAG) 532-nm laser focused on a spot size of approximately 1-μm-diameter via a microscope. The light source yielded a penetration depth of approximately 0.1 to 0.7 μm in silicon^55^. An Olympus (100 × NA = 0.9) objective lens was used for laser focusing and Raman signal collection. As shown in Fig. 6(a), the stress-free silicon surface and the remainder of the stressed silicon region were organized into voxels. Large stress values were observed only at the notch tip and nanotexture bottoms. The angled wall of the V-notch and nanotexture pillars was nearly stress-free, and each of them was covered by a single voxel. The obtained Raman spectrum consisted of a very large volume of information from the stress-free silicon surface; this information was separated from the information derived from the stressed silicon regions. Two curve-fitting approximations using a Lorentzian function^50^ were applied to separate the non-stressed spectra from the stressed Raman spectra, as shown in supplementary Figs. S6(a–d). The curve fitting performed using the Lorentz function was determined using equation (5)^56^:
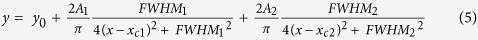
where *y*_*0*_, *A*, *FWHM*, and *x*_*c*_ are the offset, area, full-width at half-maximum, and centre (*x*-coordinate of the peak), respectively, derived from the Raman spectrum; *A*_*1*_, *FWHM*_*1*_, and *x*_*c1*_ are the information related to the non-stressed silicon peak; and *A*_*2*_, *FWHM*_*2*_, and *x*_*c2*_ are the information related to the stressed silicon peak.

### Estimation of Weibull modulus (*m*)

The Weibull estimation is widely used in engineering to relate the scattering of measured bending strength to the probability of failure (*P*_*f*_). *P*_*f*_ was defined as *n/(q + 1)*, where *n* is the rank (number of samples with strength *σ* or lower) and *q* is the total number of samples tested. The Weibull distribution was estimated by the function shown in equation (6)^57^:
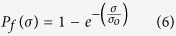

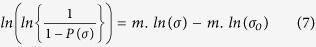


The double logarithm of equation (6) was used for ease of accessing information for the bending-strength analysis. According to the linear regression, *m* is the slope of the graph plotted from equation (7). A large value for *m* is desirable because it represents less scattering in the measured bending-strength values and more predictable failure behavior.

## Additional Information

**How to cite this article**: Kashyap, K. *et al.* Elimination of strength degrading effects caused by surface microdefect: A prevention achieved by silicon nanotexturing to avoid catastrophic brittle fracture. *Sci. Rep.* **5**, 10869; doi: 10.1038/srep10869 (2015).

## Supplementary Material

Supplementary Information

Supplementary Information

## References

[b1] Qu R. T. & Zhang Z. F. A universal fracture criterion for high-strength materials. Sci. Rep. 3, 1117; 10.1038/srep01117 (2013).

[b2] Furukawa A. & Tanaka H. Inhomogeneous flow and fracture of glassy materials. Nature Materials 8, 601–609 (2009). 10.1038/nmat2468 19525951

[b3] Eberhart M. E. Why things break. Sci Am 281, 44–51 (1999).

[b4] Fractures in metals and brittle materials. Nature 138, 132–133 (1936).

[b5] Berry J. P. Brittle fracture. Nature 212, 20 (1966).

[b6] Yokobori T., Hamamoto H. & Otsuka A. Stress condition for brittle fracture of mild steel. Nature 181, 1719–1720 (1958).

[b7] Kermode J. R. *et al.* Low-speed fracture instabilities in a brittle crystal. Nature 455, 1224–1228 (2008).

[b8] Connally J. A. & Brown S. B. Slow crack-growth in single-crystal silicon. Science 256, 1537–1539 (1992). 17836320 10.1126/science.256.5063.1537

[b9] Deegan R. D. *et al.* Wavy and rough cracks in silicon. Phys Rev E 67, 066209; 10.1103/PhysRevE.67.066209 (2003). 16241328

[b10] Hu S. M. Stress-related problems in silicon technology. J Appl Phys 70, R53–R80 (1991).

[b11] Datskos P. G., Rajic S. & Datskou I. Photoinduced and thermal stress in silicon microcantilevers. Appl Phys Lett 73, 2319–2321 (1998).

[b12] Beuth J. L. Cracking of thin bonded films in residual tension. Int J Solids Struct 29, 1657–1675 (1992).

[b13] Nam K. H., Park I. H. & Ko S. H. Patterning by controlled cracking. Nature 485, 221–224 (2012). 22575963 10.1038/nature11002

[b14] Pei Z. J., Fisher G. R. & Liu J. Grinding of silicon wafers: A review from historical perspectives. Int J Mach Tool Manu 48, 1297–1307 (2008).

[b15] Nanz G. & Camilletti L. E. Modeling of chemical-mechanical polishing - a review. Ieee T Semiconduct M 8, 382–389 (1995).

[b16] Kumagai M. *et al.* Advanced dicing technology for semiconductor wafer - Stealth dicing. Ieee T Semiconduct M 20, 259–265 (2007).

[b17] Kermode J. R. *et al.* Macroscopic scattering of cracks initiated at single impurity atoms. Nat Commun 4, 2441; 10.1038/ncomms3441 (2013). 24026345

[b18] Hu S. M. Dislocation pinning effect of oxygen-atoms in silicon. Appl Phys Lett 31, 53–55 (1977).

[b19] Alpass C. R., Murphy J. D., Falster R. J. & Wilshaw P. R. Nitrogen diffusion and interaction with dislocations in single-crystal silicon. J Appl Phys 105, 013519; 10.1063/1.3050342 (2009).

[b20] Yonenaga I. Nitrogen effects on generation and velocity of dislocations in Czochralski-grown silicon. J Appl Phys 98, 023517; 10.1063/1.1990259 (2005).

[b21] Wang G. *et al.* Mechanical strength of nitrogen-doped silicon single crystal investigated by three-point bending method. Physica B 308, 450–453 (2001).

[b22] Chen J. H. *et al.* Influence of germanium doping on the mechanical strength of Czochralski silicon wafers. J Appl Phys 103, 123521; 10.1063/1.2943272 (2008).

[b23] Huang X. M., Sato T., Nakanishi M., Taishi T. & Hoshikawa K. High strength Si wafers with heavy B and Ge codoping. Jpn J Appl Phys 2 42, L1489–L1491 (2003).

[b24] Wolf S. & Tauber R. N. Silicon processing for the vlsi era: process technology. (Lattice Press, 2000).

[b25] Yasutake K., Iwata M., Yoshii K., Umeno M. & Kawabe H. Crack healing and fracture strength of silicon-crystals. J Mater Sci 21, 2185–2192 (1986).

[b26] Kim J. M., Choi J. Y., Cho H. J., Lee H. W. & Yoo H. D. Behavior of thermally induced defects in heavily boron-doped silicon crystals. Jpn J Appl Phys 1 40, 1370–1374 (2001).

[b27] Evans A. G. Perspective on the development of high-toughness ceramics. J Am Ceram Soc 73, 187–206 (1990).

[b28] Kovar D., Thouless M. D. & Halloran J. W. Crack deflection and propagation in layered silicon nitride boron nitride ceramics. J Am Ceram Soc 81, 1004–1012 (1998).

[b29] Chen Y. L., Liu B., Huang Y. & Hwang K. C. Fracture toughness of carbon nanotube-reinforced metal- and ceramic-matrix composites. J Nanomater, 746029; 10.1155/2011/746029 (2011).

[b30] Tyagi S., Lee J. Y., Buxton G. A. & Balazs A. C. Using nanocomposite coatings to heal surface defects. Macromolecules 37, 9160–9168 (2004).

[b31] Gupta N., Priya S., Islam R. & Ricci W. Characterization of mechanical and electrical properties of epoxy-glass microballoon syntactic composites. Ferroelectrics 345, 1–12 (2006).

[b32] Chen C. N. *et al.* Strengthening for sc-Si solar cells by surface modification with nanowires. J Microelectromech S 20, 549–551 (2011).

[b33] Suwito W., Dunn M. L., Cunningham S. J. & Read D. T. Elastic moduli, strength, and fracture initiation at sharp notches in etched single crystal silicon microstructures. J Appl Phys 85, 3519–3534 (1999).

[b34] Gomez F. J. & Elices M. A fracture criterion for blunted V-notched samples. Int J Fracture 127, 239–264 (2004).

[b35] Gogotsi G. A. Fracture toughness of ceramics and ceramic composites. Ceram Int 29, 777–784 (2003).

[b36] Kim B. C. *et al.* Guided fracture of films on soft substrates to create micro/nano-feature arrays with controlled periodicity. Sci. Rep. 3, 3027; 10.1038/srep03027 (2013). 24149668 PMC3805969

[b37] Park S. *et al.* Flexible molecular-scale electronic devices. Nat Nanotechnol 7, 438–442 (2012). 22659606 10.1038/nnano.2012.81

[b38] Ko H. C. *et al.* A hemispherical electronic eye camera based on compressible silicon optoelectronics. Nature 454, 748–753 (2008). 18685704 10.1038/nature07113

[b39] Khang D. Y., Jiang H. Q., Huang Y. & Rogers J. A. A stretchable form of single-crystal silicon for high-performance electronics on rubber substrates. Science 311, 208–212 (2006). 16357225 10.1126/science.1121401

[b40] Peng K. Q., Yan Y. J., Gao S. P. & Zhu J. Synthesis of large-area silicon nanowire arrays via self-assembling nanoelectrochemistry. Adv Mater 14, 1164–1167 (2002).

[b41] Hsieh C. M., Chyan J. Y., Hsu W. C. & Yeh J. A. Fabrication of wafer-level antireflective structures in optoelectronic applications. 2007 Ieee/Leos International Conference on Optical Mems and Nanophotonics , 185–186; 10.1109/OMEMS.2007.4373902 (2007).

[b42] Designation E855-08 , ASTM, Philadelphia, PA, USA (2009).

[b43] Goto H., Saito H., Fujinami M. & Shirai H.; Toshiba Ceramics co., Ltd., Tokyo (JP). Wafer defect measuring method and apparatus. United States patent US 6,734,960. 2004 May 11.

[b44] Pei Z. J., Billingsley S. R. & Miura S. Grinding induced subsurface cracks in silicon wafers. Int J Mach Tool Manu 39, 1103–1116 (1999).

[b45] Pei Z. J. A study on surface grinding of 300 mm silicon wafers. Int J Mach Tool Manu 42, 385–393 (2002).

[b46] Zarudi I. & Zhang L. Subsurface damage in single-crystal silicon due to grinding and polishing. J Mater Sci Lett 15, 586–587 (1996).

[b47] Pilkey W. D. & Pilkey D. F. Peterson’s stress concentration factors. (Wiley, 2008).

[b48] Namazu T., Isono Y. & Tanaka T. Evaluation of size effect on mechanical properties of single crystal silicon by nanoscale bending test using AFM. J Microelectromech S 9, 450–459 (2000).

[b49] Cook R. F. Strength and sharp contact fracture of silicon. J Mater Sci 41, 841–872 (2006).

[b50] Komatsubara M. *et al.* Raman spectrum curve fitting for estimating surface stress distribution in single-crystal silicon microstructure. Jpn J Appl Phys 48, 04C021; 10.1143/Jjap.48.04c021 (2009).

[b51] Parker J. H., Feldman D. W. & Ashkin M. Raman scattering by silicon and germanium. Phys Rev 155, 712–714 (1967).

[b52] Bohm C., Hauck T., Juritza A. & Muller W. H. Weibull statistics of silicon die fracture. El Packag Tech Conf , 782–786; 10.1109/EPTC.2004.1396714 (2004).

[b53] Kendall K., Alford N. M., Tan S. R. & Birchall J. D. Influence of toughness on Weibull modulus of ceramic bending strength. J Mater Res 1, 120–123 (1986).

[b54] Bogdanowicz J. *et al.* Non-destructive characterization of saw damage in silicon photovoltaics substrates by means of photomodulated optical reflectance. Phys Status Solidi C 9, 2116–2119 (2012).

[b55] Lee N. *et al.* High contrast scanning nano-Raman spectroscopy of silicon. J Raman Spectrosc 38, 789–796 (2007).

[b56] Gough W. Graphical analysis of a Lorentzian function and a differentiated Lorentzian function. J Phys Pt a Gen 1, 704–709 (1968).

[b57] Klein C. A. Characteristic strength, Weibull modulus, and failure probability of fused silica glass. Opt Eng 48, 113401; 10.1117/1.3265716 (2009).

