# Supplementary material for: Elimination of strength degrading effects caused by surface microdefect: A prevention achieved by silicon nanotexturing to avoid catastrophic brittle fracture
Source: Sci Rep. 2015 Jun 4;5:10869. doi: 10.1038/srep10869 (PMC4455193; doi:10.1038/srep10869)
Supplement: Supplementary Information [file srep10869-s1.pdf]

Supporting information:

Elimination of strength degrading effects caused by surface microdefect: A prevention achieved by silicon nanotexturing to avoid catastrophic brittle fracture

Kunal Kashyap[1], Amarendra Kumar[1], Chuan-Torng Huang[1], Yu-Yun Lin[2], Max T. Hou[3] and J. Andrew Yeh[1,4,5,*]

[1]Institute of Nanoengineering and Microsystems, National Tsing Hua University, No. 101, Section 2, Kuang-Fu Road, Hsinchu 30013, Taiwan.

[2]Department of Civil Engineering, National Cheng Kung University, No.1, University Road, Tainan City 701, Taiwan.

[3]Department of Mechanical Engineering, National United University, No.1, Lienda, Miaoli 36003, Taiwan.

[4]Department of Power Mechanical Engineering, National Tsing Hua University, No. 101, Section 2, Kuang-Fu Road, Hsinchu 30013, Taiwan.

[5]Instrument Technology Research Center, National Applied Research Laboratories, 20, R&D Road VI, Hsinchu Science Park, Hsinchu 30076, Taiwan.

*Correspondence and requests for materials should be addressed to J.A.Y. (e-mail:jayeh@mx.nthu.edu.tw).

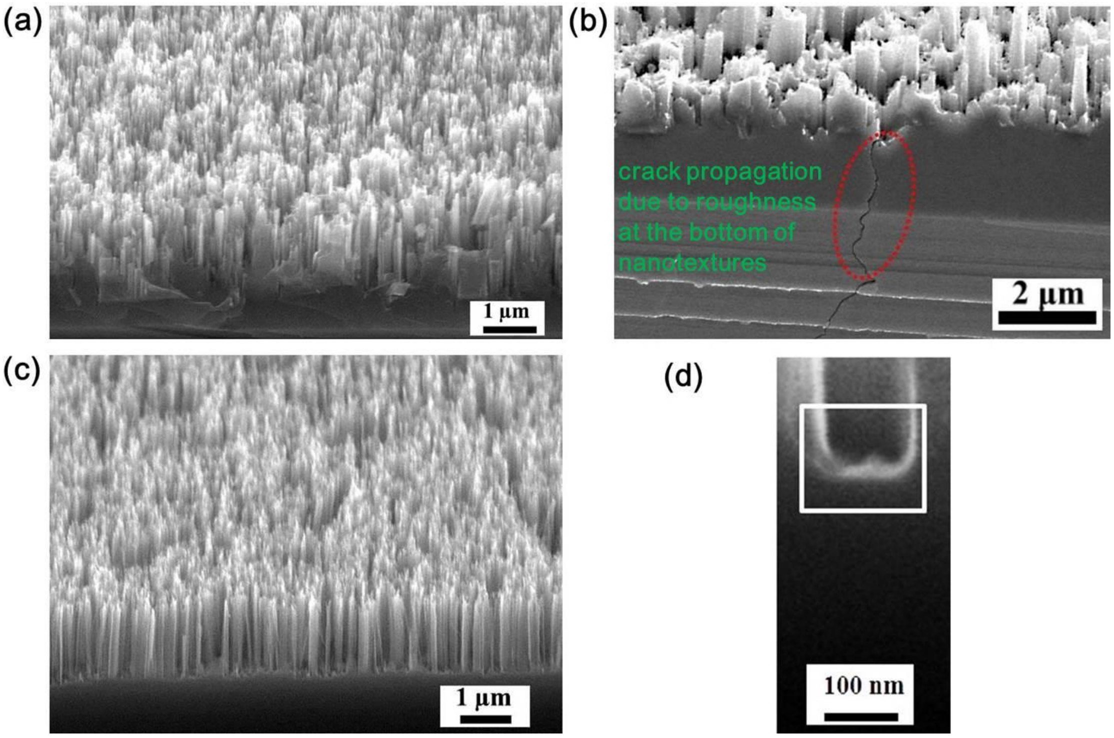

Figure S1. Morphological dependence of silicon nanotexturing on strength and fracture. (a) Silicon nanotextures consisting large roughness at their bottom (b) Fracturing of the sample due to roughness on the bottom of nanotextures (c) Silicon nanotextures fabricated with smoother morphology at their bottom (d) 100-nm-wide nanotexture nearly without roughness.

Silicon nanotexturing was performed by wet chemical etching process. Fabrication of silicon nanotextures was controlled to achieve the maximum possible smooth morphology at their bottom by tunning the etchant of metal assisted etching. The amount of oxidizing agent was increased to form the uniform silicon dioxide beneath the Ag particle acting as a catalyst for nanotextures formation. The HF content was kept low which reduces the overall etching rate and the etching was less

aggressive[1].

The silicon nanotextures with large roughness (as shown in Fig. S1(a) and (b)) concentrate the stress because of the presence of sharp corners and reduces the strength. The existence of large stress concentration triggers the crack and fracture the sample. Due to improper morphology, the intentionally fabricated nanotextures for distributing the concentrated stress from the pre-existing defects act as the dominant defect, which is undesirable. The maximum possible smoother morphology of the nanotextures bottom is required to intensify the stress redistribution, therefore strengthen the samples.

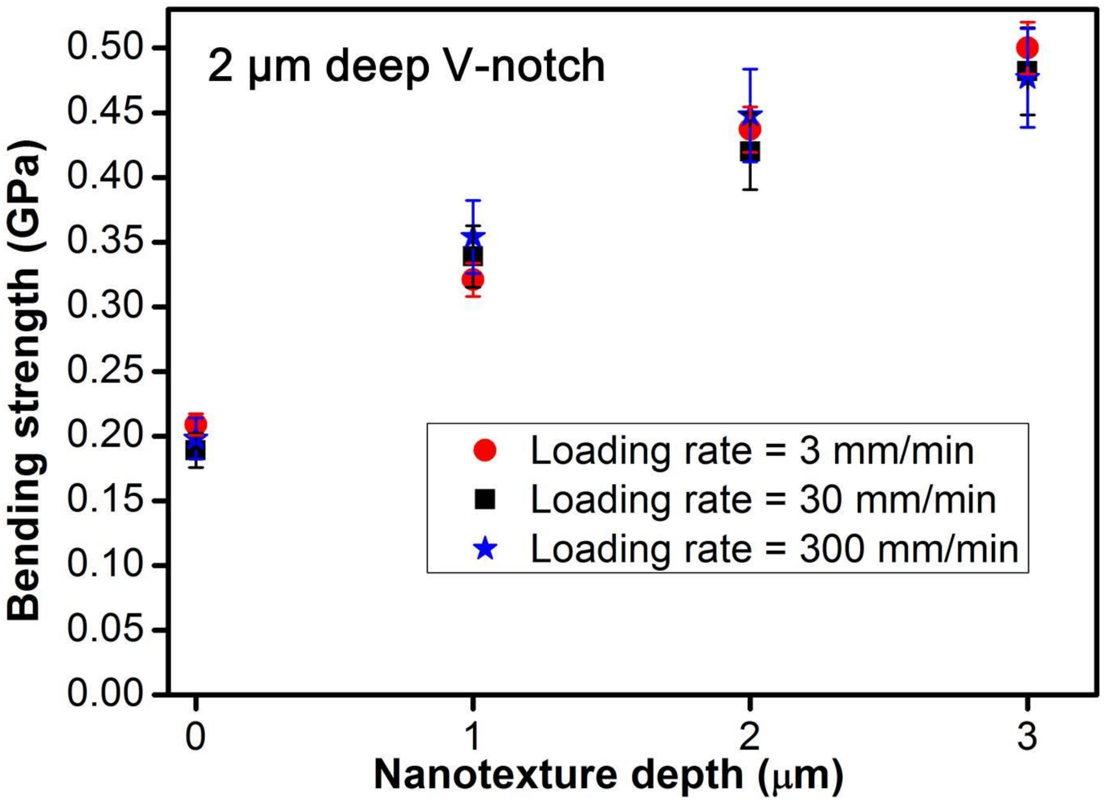

Figure S2. Effect of loading rate on bending strength enhancement of the V-notched samples with nanotexturing. All the three point bending test (3PB) discussed in Fig. 3 was performed at a loading rate of 30 mm/min. We further extended our study to evaluate the 3PB tests for 2 μm deep non-textured and nanotextured samples at a lower loading rate of 3 mm/min and at a higher loading rate of 300 mm/min. The bending strength of non-textured and nanotextured shows negligible variations after changing the loading rate. The extent of bending strength enhancement of the V-notched samples with nanotexturing was also unaltered by varying the loading rate. The loading rate will not affect the bending strength of the brittle materials unless it shows the dynamic effect[2]. The strength of silicon is generally evaluated under quasistatic conditions. The dynamic effect in silicon can

only be observed under impulsive loading i.e. extremely high loading rate. When the deformation energy approaches the kinetic energy then the dynamic effect can be observed, which is generally been avoided while determining the bending strength of silicon[3]. The below equation 1 confirms that dynamic effect can be only observed under impulsive loading[3] for silicon.

$$\frac{E_k}{U} = \left(\Lambda \frac{W\dot{\Delta}(t)}{c_0 \Delta(t)}\right)^2 \quad (1)$$

where E_k, U, W, $\dot{\Delta}(t)$, $\Delta(t)$, c_0, and Λ are kinetic energy, deformation energy, specimen width, displacement rate, load line displacement, longitudinal wave speed (i.e., the speed of sound) in a one-dimensional bar of silicon (8490 m/s)[4], and geometry factor. The ratios of kinetic energy to deformation energy is extremely small for the loading rate of 3 mm/min, 30 mm/min and 300 mm/min. These loading rates satisfies the quasistatic condition for determining the bending strength and validate the fact for not affecting the bending strength under variable loading rate.

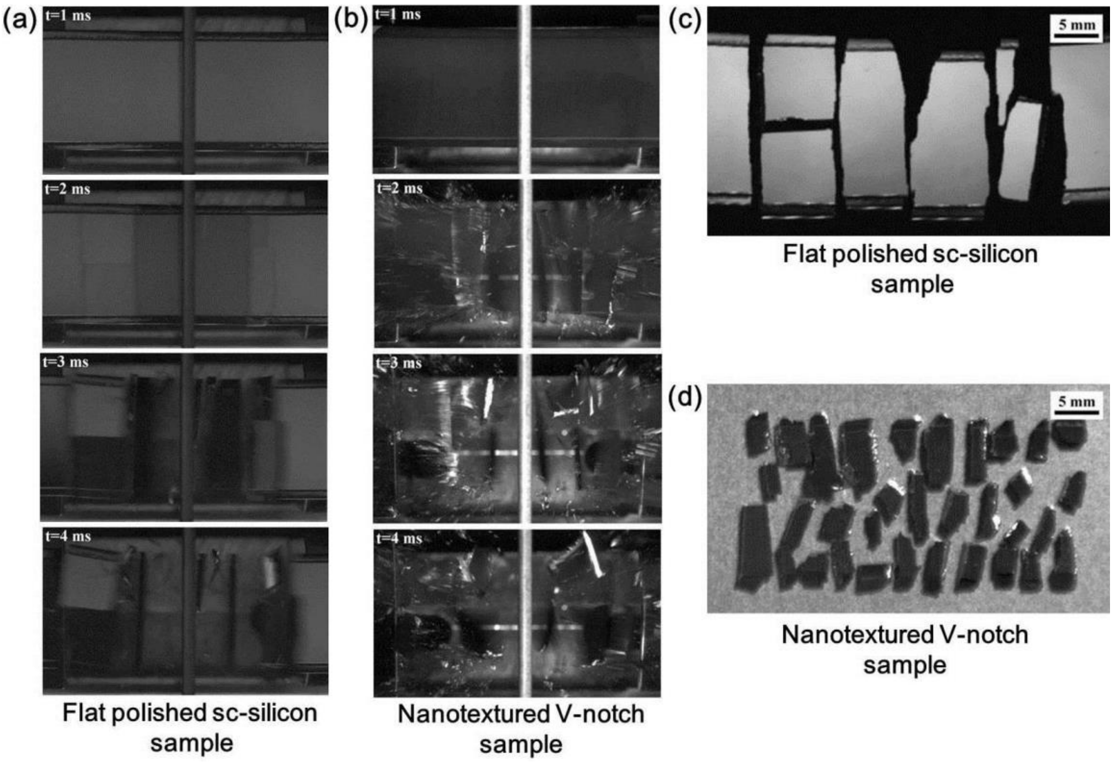

Figure S3. Comparison of fragmentation during fracture for a flat polished sc-silicon sample and 7-μm-deep nanotextured (depth of approximately 10.5 μm) V-notch sample.(a) and (c) Flat polished sc-silicon sample composed of fewer fragments just after a fracture. (b) and (d) Nanotextured V-notch sample composed of multiple fragments just after a fracture. The nanotextured sample in (b) sustains a larger force prior to fracture compared to the flat polished sc-silicon sample in (a), demonstrating the improved strength of the silicon substrate with nanotexturing.

The dynamic response of the fracture in the 3PB test was observed using a high speed camera (IDT Y-4), and was recorded at a frame rate of 1000 frames/sec under the illumination of a 500-W halogen lamp. For any type of brittle material, the greater the number of fragments produced during fracture under applied load, the larger will

be the sample strength due to high strain energy absorption. The elastic strain energy stored in the sample at failure is converted into surface energy and kinetic energy of dynamic fracture after failure as shown in equation (2)[5].

$$\frac{\sigma_f^2}{2E}V \rightarrow 2\gamma A + U_K \tag{2}$$

Bending strength (σ_f) increases with surface area (A) / volume (V) which explains more fragmentation have higher bending strength, where E is Young's modulus, γ is surface energy density and U_K is the kinetic energy of dynamic fracture. A larger number of fragments of polished sc-silicon samples compared to non-textured V-notch samples (only two fragments as shown in Fig. 4 of the main article) and relatively lesser number of fragments of polished sc-silicon samples compared to nanotextured V-notch samples also reveal the strength degradation due to V-notch and recovery of strength even more than the flat polished sc-silicon samples due to nanotexturing.

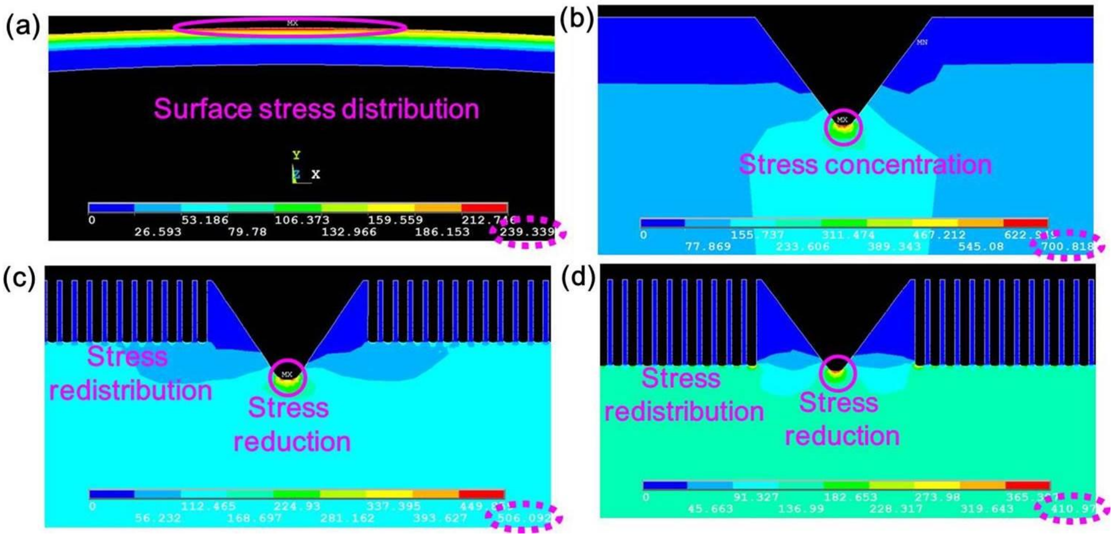

Figure S4. FEA simulation results of the 3PB model demonstrating the effect of nanotexturig on stress distribution. All the samples are simulated under same applied force. (a) Stress distribution for sc-silicon sample without V-notch and nanotexturing (b) Stress distribution for non-textured 2-µm-deep V-notch sample (c) Stress distribution for 2-µm-deep V-notch sample with 1 µm depth of nanotexturing (d) Stress distribution for 2-µm-deep V-notch sample with 2 µm depths of nanotexturing.

The flat sc-silicon sample shows the uniform distribution of the stress on all over the surface, but stress is concentrated at the V-notch tip and signifies the degradation of strength with compare to flat sc-silicon sample. The nanotexturing enables the stress redistribution and reduces the maximum value of stress, resulting in the recovery of strength.

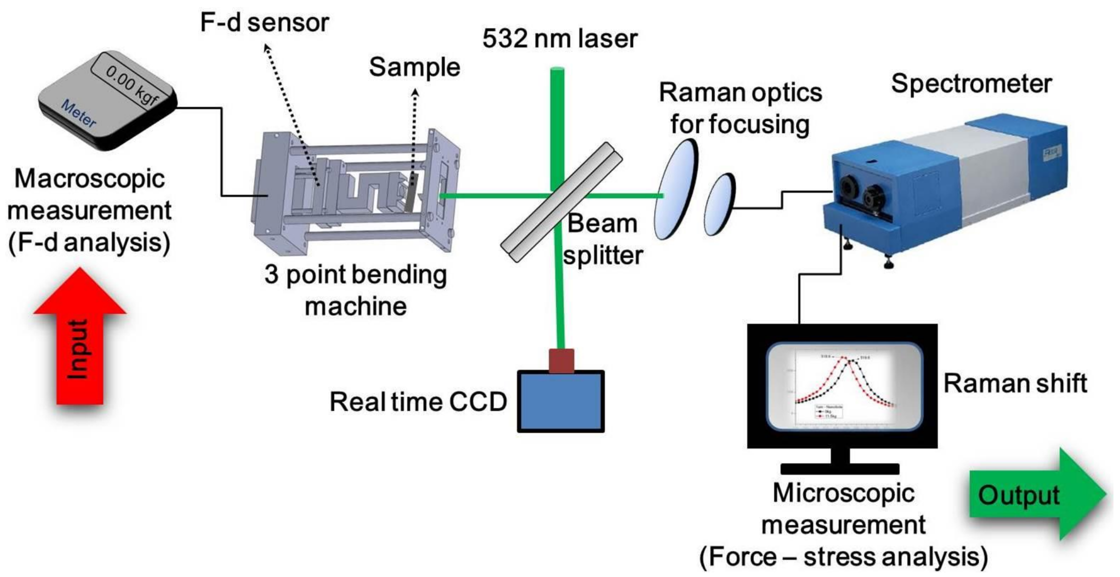

Figure S5. Experimental set up for micro-Raman measurement.

Macroscopic measurement performed by 3PB machine was coupled with the microscopic measurement performed by micro-Raman spectroscopy. The information of force-displacement was connected with the corresponding stress to confirm the stress redistribution at notch tip and nearby nanotexture region.

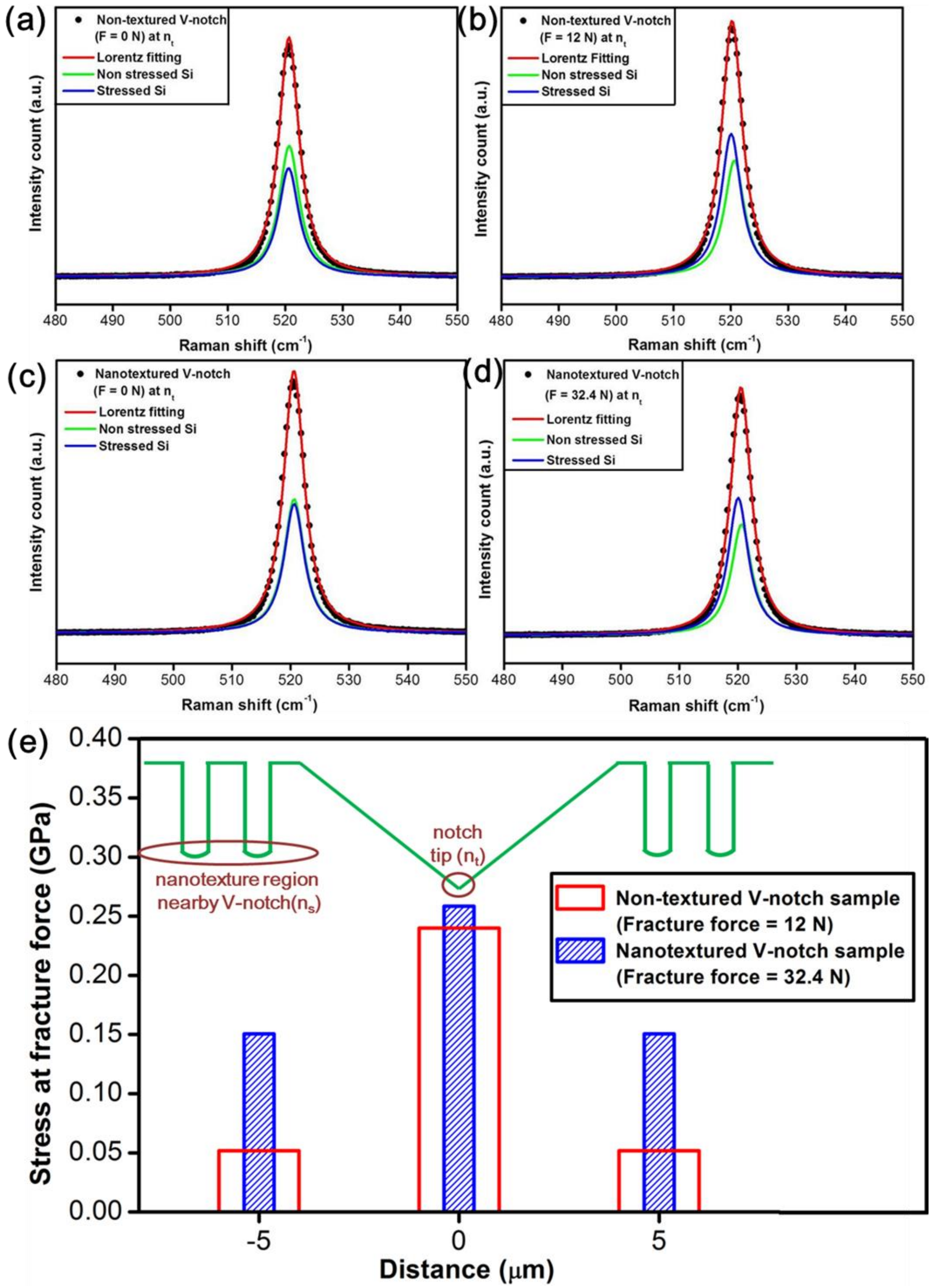

Figure S6. Micro-Raman analysis of a 7-μm-deep V-notch sample using Lorentzian curve fitting, which elucidates the mechanism of stress redistribution. (a) and (c) Raman spectra with no applied load for non-textured and nanotextured

V-notch samples, respectively. Both stressed and non-stressed silicon peaks remain at approximately 520.6 cm^{-1}. (b) The Raman spectrum of the non-textured V-notch sample with 12 N of applied force. The non-stressed silicon peak remains at approximately 520.6 cm^{-1} while the stressed silicon peak exhibits a shift of 0.53 cm^{-1} towards lower wavenumbers. (d) Raman spectrum for the nanotextured V-notch sample with 32.4 N of applied force. The non-stressed silicon peak remains at approximately 520.6 cm^{-1} while the stressed silicon peak exhibits a shift of 0.55 cm^{-1} towards lower wavenumbers. (e) A graph summarizing the results of micro-Raman analysis.

With no load applied, both non-textured and nanotextured V-notch samples of 7-μm-depth measured at the notch tip retain a peak at approximately 520.6 cm^{-1}, as shown in Figs. S6(a) and (c), which is similar to the reported value for the non-stressed silicon peak[6]. Under the 3PB condition, one peak shifted by 0.53 cm^{-1} to lower wave numbers at the applied load of 12 N for the non-textured V-notch sample and by 0.55 cm^{-1} at 32.4 N for the nanotextured V-notch sample, as shown in Figs. S6(b) and (d), respectively. This reflects changes in the spectral information of the stressed silicon region with respect to the other peak that remains at approximately 520.6 cm^{-1} for both samples, which represents the spectral information of the stress-free silicon surface.

Non-textured V-notch samples endured fracture at a force of 12 N, but nanotextured V-notch samples sustained a larger force of 32.4 N while generating nearly the same amount of stress (approximately 0.26 GPa) at the notch tip. Just prior to fracture, the nanotextured V-notch samples exhibited 0.15 GPa of stress in the region nearby the V-notch (n_s), which is 3 fold larger compared to the non-textured V-notch samples exhibiting 0.05 GPa of stress in n_s, indicating the reduction of effective defect size and stress redistribution at the notch tip and in the nanotextured region.

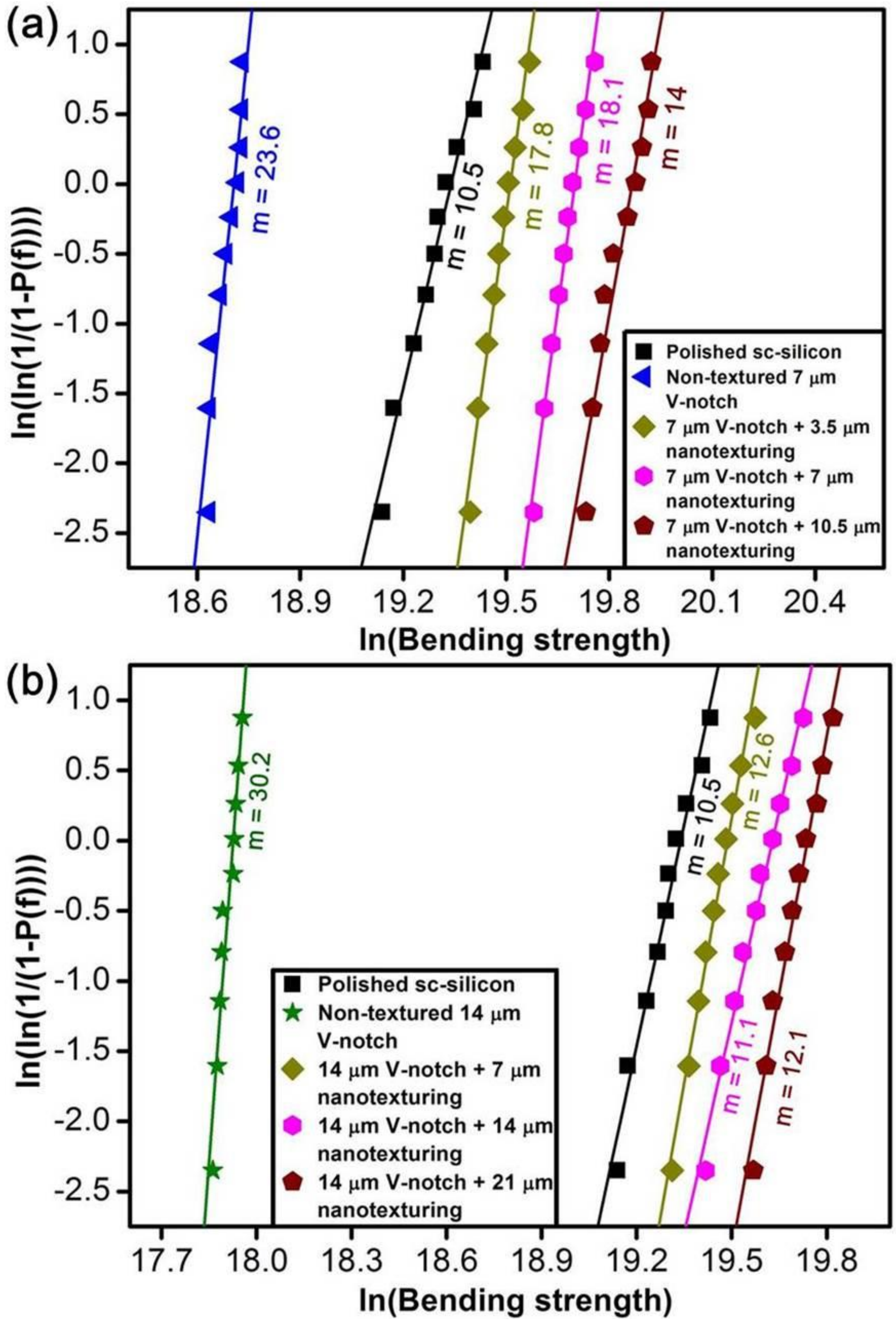

Figure S7. Weibull analysis for explaining the strengthening and the reliability of nanotexturing methodology. (a) Strength and reliability enhancement in

comparison with polished sc-silicon samples and nanotexturing of 7-μm-deep V-notch samples. (b) Strength and reliability enhancement in comparison with polished sc-silicon samples and nanotexturing of 14-μm-deep V-notch samples.

The nanotexturing not only recovers and enhances the bending strength, but also increases the Weibull modulus (m) compared to the polished sc-silicon samples as shown in Fig. S7(a) and (b). The Weibull modulus (m) measured for 3.5 μm, 7 μm, and 14 μm of nanotexturing the 7 μm of V-notch samples was 17.8, 18.1, and 14, respectively. The Weibull modulus (m) measured for 7 μm, 14 μm, and 21 μm of nanotexturing the 14 μm of V-notch samples was 12.6, 11.1, and 12.1, respectively. The Weibull modulus decreases with the depth of nanotexturing, because the occurrence of non-uniformity in the morphology of deeper nanotextures. Even though, the nanotextured samples maintain the larger value of ‘m’ compared to polished sc-silicon sample for deeper nanotexturing.

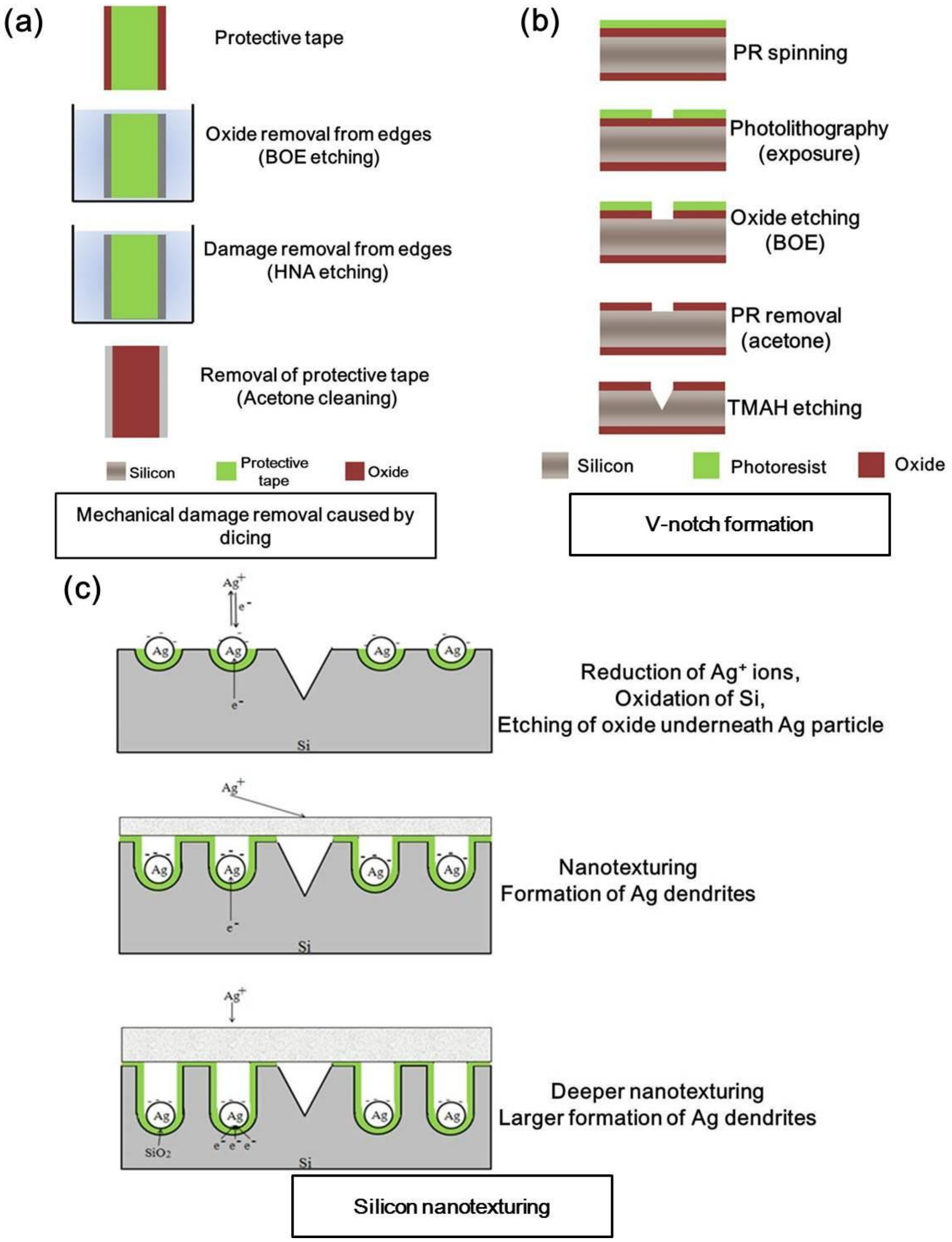

Figure S8. Detailed fabrication process flow. (a) Mechanical damage removal caused by sample dicing. (b) Fabrication of anisotropically etched V-notch. (c) Fabrication of nanotexturing the V-notch samples

References

1 Huang, Z. P., Geyer, N., Werner, P., de Boor, J. & Gosele, U. Metal-Assisted Chemical Etching of Silicon: A Review. *Adv Mater* **23**, 285-308 (2011).

2 Liu, C., Knauss, W. G. & Rosakis, A. J. Loading rates and the dynamic initiation toughness in brittle solids. *Int J Fracture* **90**, 103-118 (1998).

3 Anderson, T. L. *Fracture Mechanics: Fundamentals and Applications, Second Edition*. Chapter 4, 173-177 (Taylor & Francis, 1994).

4 Laurell, T. & Lenshof, A. *Microscale Acoustofluidics*. Chapter 6, 105 (Royal Society of Chemistry, 2014).

5 Cook, R. F. Strength and sharp contact fracture of silicon. *J Mater Sci* **41**, 841-872 (2006).

6 Parker, J. H., Feldman, D. W. & Ashkin, M. Raman Scattering by Silicon and Germanium. *Phys Rev* **155**, 712-714 (1967).
